# Influence of genetic polymorphisms in P2Y12 receptor signaling pathway on antiplatelet response to clopidogrel in coronary heart disease

**DOI:** 10.1186/s12872-022-02988-w

**Published:** 2022-12-30

**Authors:** Yan-Jiao Zhang, Dong-Jie Li, Zhong-Yi Li, Xiao-Lei Hu, He Li, Qi-Lin Ma, Xiao-Ping Chen

**Affiliations:** 1 Anhui Province Maternity & Child Health Hospital, Hefei, 230000 Anhui People’s Republic of China; 2 Department of Clinical Pharmacology, Xiangya Hospital, Central South University, Changsha, 410008 Hunan People’s Republic of China; 3 Institute of Clinical Pharmacology, Central South University, Hunan Key Laboratory of Pharmacogenetics, Changsha, 410078 Hunan People’s Republic of China; 4 Department of Urology, Xiangya Hospital, Central South University, Changsha, 410008 People’s Republic of China; 5 National Clinical Research Center for Geriatric Disorders, Changsha, 410008 People’s Republic of China; 6 Department of Urology, Xiangya Hospital, Central South University, Changsha, 410008 Hunan People’s Republic of China; 7 Hunan Cancer Hospital, The Affiliated Cancer Hospital of Xiangya School of Medicine, Central South University, Changsha, 410013 People’s Republic of China; 8 Department of Cardiovascular Medicine, Xiangya Hospital, Central South University, Changsha, 410008 Hunan People’s Republic of China

**Keywords:** Genetic polymorphisms, P2Y12, Coronary heart disease

## Abstract

**Backgrounds:**

Remarkable interindividual variability in clopidogrel response is observed, genetic polymorphisms in P2RY12 and its signal pathway is supposed to affect clopidogrel response in CHD patients.

**Methods:**

539 CHD patients treated with clopidogrel were recruited. The platelet reaction index (PRI) indicated by VASP-P level were detected in 12–24 h after clopidogrel loading dose or within 5–7 days after initiation of maintain dose clopidogrel. A total of 13 SNPs in relevant genes were genotyped in sample A (239 CHD patients). The SNPs which have significant differences in PRI will be validated in another sample (sample B, 300 CHD patients).

**Results:**

CYP2C19*2 increased the risk of clopidogrel resistance significantly. When CYP2C19*2 and CYP2C19*3 were considered, CYP2C19 loss of function (LOF) alleles were associated with more obviously increased the risk of clopidogrel resistance; P2RY12 rs6809699C > A polymorphism was also associated with increased risk of clopidogrel resistance (AA vs CC: *P* = 0.0398). This difference still existed after stratification by CYP2C19 genotypes. It was also validated in sample B. The association was also still significant even in the case of stratification by CYP2C19 genotypes in all patients (sample A + B).

**Conclusion:**

Our data suggest that P2RY12 rs6809699 is associated with clopidogrel resistance in CHD patients. Meanwhile, the rs6809699 AA genotype can increase on-treatment platelet activity independent of CYP2C19 LOF polymorphisms.

**Supplementary Information:**

The online version contains supplementary material available at 10.1186/s12872-022-02988-w.

## Introduction

Atherosclerosis thrombosis can lead to the development of acute coronary syndrome (ACS) and acute myocardial infarction, which is a severe threat to human health. The number of deaths caused by coronary atherosclerosis alone accounts for one-seventh of all-cause deaths worldwide [1]. Because the platelet activation plays an essential role in the formation of thrombus in atherosclerosis thrombosis, antiplatelet therapy has established as a cornerstone in the treatment of coronary heart disease (CHD). Clopidogrel, a P2Y12 receptor antagonist, is recommended to be widely used in patients suffered from acute coronary syndrome (ACS) and post percutaneous coronary intervention (PCI) to prevent future thrombotic events. However, the evidence shows that about 5–44% of patients treated with the standard dose of clopidogrel failed to display an adequate antiplatelet aggregation response [2]. As a result, patients with clopidogrel resistance (CR) may show an increased risk of recurrent adverse cardiovascular events [3]. The variability in clopidogrel response is explained by multiple independent factors including genetic polymorphisms [4].

Clopidogrel is a prodrug that requires two steps of bioactivation via cytochromes P450 (CYP) to form the active thiol derivative. CYP2C19 plays a crucial role in its bioactivation. Genetic polymorphisms that result in remarkable interindividual variability in CYP2C19 activity have been observed. Especially, the *CYP2C19* loss-of-function (LOF) variants, such as CYP2C19*2 and CYP2C19*3, can decrease the AUC of the clopidogrel active metabolite, and patients carrying these variant alleles show higher on-treatment platelet activity and increased risk of atherothrombotic events [5–7]. The American Food and Drug Administration (FDA) even announced a black boxed warning on clopidogrel about CYP2C19*2 and CYP2C19*3.

The active thiol derivative metabolite of clopidogrel acts through competing with the soluble platelet agonist adenosine 5-diphosphate (ADP) for the platelet P2Y12 receptor. The inhibition of the P2Y12 receptor will lead to the inhibition of the integrin glycoprotein IIb/IIIa (GPIIb/IIIa) complex on the platelet surface, which is called integrin “inside-out” signaling process [8]. Activation of the integrin αIIbβ3 stimulates platelet adhesion and aggregation and triggers “outside-in” signaling, resulting in platelet spreading, additional granule secretion, stabilization of platelet adhesion and aggregation, and clot retraction [9]. Several proteins are involved in the P2Y12-integrin αIIbβ3 activation pathway. Upon P2Y12 activation, the P2Y12-coupled Gi protein can activate phosphatidylinositol-3-kinase (PI3K, encoded by *PIK3CA*) in platelets, which in turn activates the small GTPase Rap1, a critical mediator of integrin GPIIb/IIIa activation [10–12]. Calcium and diacylglycerol-guanine nucleotide exchange factor 1 (CalDAG-GEF1) is responsible for the conversion of Rap1 from the inactive GDP-bound form to the active GTP-bound form, the latter could interact with the Rap1-GTP-interacting adaptor molecule (RIAM) [13]. Talin, encoded by the *TLN1* gene, is a ∼ 270 kDa cytoskeleton adaptor protein contains a globular head region that directly links β-integrin. The binding of talin with integrin is the necessary final step for integrin activation [14]. While RIAM, encoded by the gene *APBB11P,* functions as a scaffold that connects the membrane targeting sequence in Rap1 to talin, thereby recruiting talin to the plasma membrane and activating integrins [15]. A study on an inherited platelet disorder in siblings using whole-exome sequencing has identified a culprit mutation (cG742T) in *RASGRP2*, the gene coding for CalDAG-GEFI, to be causative [16]. Platelets from individuals with the mutation showed reduced ability to activate Rap1 and improper αIIbβ3 integrin inside-out signaling [16]. The aIIb subunit (GPIIb) and the β3 (GPIIIa) are encoded by *ITGA2B* and *ITGB3*, respectively. Single nucleotide polymorphisms (SNPs) in *ITGA2B* and *ITGB3* were found to be associated with indexes of platelet and coagulation hemostasis in healthy Chinese people [17]. Similarly, our previous study in healthy Chinese subjects has also demonstrated that the *ITGA2B* rs5911 polymorphism can increase the effect of ticagrelor on ADP-induced platelet aggregation [18]. Moreover, the associations between polymorphisms *P2RY12* polymorphisms (T744C, G52T) and platelet response are also reported [19, 20].

A study has shown that the use of P2Y12 inhibitor monotherapy, as an alternative approach to DAPT, in patients undergoing coronary revascularization. P2Y12 inhibitor monotherapy was associated with similar risks of death, myocardial infarction, or stroke and lower risks of major bleeding compared with DAPT [21].

However, there was a paucity of studies on other genes in platelet related to the P2Y12 receptor signaling pathway and clopidogrel response. Hence, our study was designed to elucidate the degree of crucial genetic polymorphisms related to the P2Y12 receptor signaling pathway on the clopidogrel resistance in Chinese CHD patients.

## Materials and methods

### Study subjects

A total of 539 consecutive CHD patients treated with clopidogrel from Xiangya Hospital, Central South University from September 2014 to November 2018 in this prospective clinical study. The age of the patients ranged from 18 to 80 years. These samples were divided into discovery (n = 239) and validation (n = 300) sets. All patients received dual antiplatelet therapy (DAPT) with aspirin and oral administration of 300 mg loading dose (LD) clopidogrel, or 75 mg daily maintaining dose (MD) of clopidogrel for at least 5 days. Venous blood samples were drawn in 6:00–7:00 Am 12–24 h after LD of clopidogrel or on day 5–7 after the initiation of MD of clopidogrel for analysis of platelet reaction index (PRI) and DNA extraction. Subjects were excluded if they had a history of a bleeding disorder, current warfarin use, myelodysplastic or myeloproliferative disorders, chronic liver disease or hypersensitivity to clopidogrel. Subjects were also excluded if they were pregnant, with platelet count less than 10^5^ cell/mm^3^ (thrombocytopenia), or creatinine clearance less than 25 mL/min, or prior use of GPIIb/IIIa antagonist before the procedure. Questionnaires and medical records were used to collect family and medical history, age, gender, smoking and alcohol habits, diabetic status and other disease complications, co-medications, platelet count, mean platelet volume (MPV), and physical activities. Patients were followed up by telephone interviewers using standardized questionnaires. The primary endpoint of this study was major adverse cardiac events (MACE), defined as a composite of cardiac death, myocardial infarction (MI), and repeat target vessel revascularization. The study protocols were approved by the Ethics Committee of Central South University (No. CTXY-140002-13) and followed the Declaration of Helsinki. It was also registered on the Chinese Clinical Trial Registry (http://www.chictr.org.cn) (ChiCTR-OPN-15006260). Informed consent was signed by all subjects after explanation on the aims and benefits of this research project.

### Vasodilator-stimulated phosphoprotein-phosphorylation (VASP-P) assay

PRI was detected within 24 h after blood is drawn. To avoid platelet activation induced by needle puncture, the initial first blood millimeters were discarded. Blood samples were immediately collected in a vacutainer tube containing 3.8% trisodium citrate, filled to capacity, and analyzed immediately. A standardized flow cytometric assay (Platelet VASP®; Diagnostica Stago, Biocytex, Marseille, France) was used to determine the VASP-P level in whole blood according to the standard protocols [22]. Briefly, 10 μL blood sample was incubated with PGE1 or with PGE1 + ADP for 10 min and fixed with paraformaldehyde, after which the platelets were permeabilized with non-ionic detergent. The cells were labeled with a primary monoclonal antibody against serine 239-phosphorylated VASP (16C2), followed by a secondary fluorescein isothiocyanate-conjugated polyclonal goat anti-mouse antibody. The total duration of the preparation was within 30 min after blood sampling. Analyses were then performed on EPICS XL-MCL flow cytometer (Beckman Coultronics, Margency, France). The platelet population was identified from its forward and side scatter distribution and 10,000 platelets were gated for each sample. Platelet reactivity index (PRI) was calculated from the median fluorescence intensity (MFI) of samples with the formula:

$${\text{PRI}}\;\left( \% \right) = \, [\left( {{\text{MFI}}_{{{\text{PGE}}1}} - {\text{MFI}}_{{{\text{PGE1}} + {\text{ADP}}}} /{\text{ MFI}}_{{{\text{PGE}}1}} } \right) \times 100\% .$$

### SNP selection and genotyping

Genomic DNA was purified from peripheral blood leukocytes by Wizard® Genomic DNA Purification Kit (Promega Corperation). A total of 13 SNPs in 8 genes including *P2RY12* (rs2046934, rs6809699), *PIK3CA* (rs67562832, rs67562832)*, RASGRP2* (rs2230414)*, APBB1IP* (rs11015149)*, TLN1 (*rs2295795, rs10814270*), ITGB3* (rs3785873, rs58847127), *ITGA2B* (rs3760364) and *CYP2C19* (rs4244285/CYP2C19*2, rs4986893/ CYP2C19*3) were selected in our study. The SNPs selected were either reported to be clinically relevant or htSNPs indicated by Haploview analysis (www.broad.mit.edu/mpg/haploview/index.php) with a frequency > 5% in the 1000 genomes project for 97 Chinese Han Beijing (CHB) individuals (www.1000genomes.org). Details of the SNPs were shown in Table 1. Method of polymerase chain reaction-restriction fragment length polymorphism (PCR–RFLP) was used for CYP2C19*2 and CYP2C19*3 genotyping as described previously [23]. The other SNPs were genotyped by Sequenom’s MassARRAY assay (Sequenom, San Diego, California, USA). Genotypes of 10% of the samples were verified by PCR-based sequencing. All selected SNPs were genotyped in discovery samples (239 CHD patients). The SNPs which have significant differences in PRI will be validated in validation samples(300 CHD patients).

**Table 1:** Information of 13 SNPs selected in this study

Gene	SNP	Chr	Alleles	Functional consequence	MAF
*CYP2C19*	rs4244285	10:94781859	G > A	Pro227Pro	0.221
	rs4986893	10:94780653	G > A	stop gained	0.014
*P2RY12*	rs2046934	3:151339854	G > A	intron variant	0.205
	rs6809699	3:151338810	C > A	Gly12Gly	0.089
*PIK3CA*	rs67562832	3:179173633	A > G	intron variant	0.075
	rs77576241	3:179156079	C > T	intron variant	0.051
*RASGRP2*	rs2230414	11:64728885	C > A	Gly583Gly	0.36
*APBB1IP*	rs11015149	10:26523246	C > A	intron variant	0.15
*TLN1*	rs2295795	9:35712006	G > A	Ser1227Leu	0.278
	rs10814270	9:35704153	C > T	Ala2023Ala	0.4
*ITGB3*	rs3785873	17:47301872	G > A	intron variant	0.208
	rs58847127	17:47257956	G > C	intron variant	0.142
*ITGA2B*	rs3760364	17:44390436	T > A	upstream variant	0.011

### Statistical analysis

Statistical analysis was performed using SPSS Statistics 19.0 (SPSS Inc., Chicago, USA). Continuous variables were presented as mean ± standard deviation (SD). The unpaired two-sided Student's t-test was used to compare normally distributed continuous data between two groups, and comparisons of difference in PRI among genotypes were carried out by one-way ANOVA test under the co-dominant model. The Benjamini–Hochberg procedure was performed to control the false discovery rate for multiple testing. χ2-test was used to compare categorical variables between/among groups. Unconditional logistic regression was used to assess the association between genotypes and clopidogrel resistance, odds ratios (OR) and the 95% confidence intervals (CI) were calculated and adjusted by the covariates. The Non-CR group was been defined as control group. Association analyses were conducted under three genetic models, including co-dominant, dominant, and recessive. Given D is the major allele and d is the minor allele for an SNP, the co-dominant model means DD versus Dd versus dd, the dominant model means DD versus Dd + dd, while the recessive model means DD + Dd versus dd. Statistical significance was defined as *P* < 0.05.

## Results

### Baseline characteristics of study patients and genotyping

From 2014 to 2018, a total of 539 eligible CHD patients with clopidogrel treatment were recruited in this study (Table 2). According to PRI from VASP-P assay, the patients were categorized into clopidogrel resistance (CR, PRI > 50%) and non-CR (PRI≦50%) [24, 25]. Among the patients, 351 (65.12%) were classified as CR, and 188 (34.88%) were classified as non-CR. There was no significant difference between the two groups regarding age, gender, smoking and alcohol administration habits, disease complications (diabetes, hypertension, dyslipidemia), co-medications (proton pump inhibitor, calcium channel blocker, statin, morphine), platelet count and MPV (*P* > 0.05). Meanwhile, the difference between the groups with 300 mg LD or 75 mg/d MD was not also statistically significant (*P* > 0.05, Table 3). So we combined patients with LD and MD as a whole in the subsequent analysis. Patients in the CR group showed significantly higher mean PRI value than the non-CR group (*P* = 1.0 × 10^−43^).

**Table 2:** Baseline characteristics of 539 patients in clopidogrel resistance and non-resistance groups

Parameters	Sample A (N = 239)			Sample B (N = 300)				
	Non-CR (N = 90)	CR (N = 149)	*P* value	Non-CR (N = 98)		CR (N = 202)	*P* value	All patients (N = 539)
Age (x ± SD)	61.67 ± 10.67	61.08 ± 10.02	0.669	61.87 ± 10.81		60.05 ± 9.61	0.143	60.94 ± 10.13
Male, n (%)	59 (65.6)	97 (65.1)	0.943	70 (71.4)		131 (64.9)	0.256	357 (66.2)
Diabetes, n (%)	14 (17.9)	29 (25.2)	0.234	15 (20.0)		32 (17.9)	0.691	90 (16.7)
Hypertension, n (%)	56 (71.8)	79 (66.9)	0.473	46 (61.3)		92 (51.4)	0.147	273 (50.6)
Dyslipidemia, n (%)	9 (11.4)	25 (21.7)	0.063	16 (21.3)		46 (25.7)	0.460	96 (17.8)
Smoking, n (%)	31 (39.2)	47 (37.3)	0.781	25 (34.7)		71 (40.3)	0.410	174 (32.3)
Alcohol use, n (%)	21 (28.4)	32 (28.1)	0.963	20 (27.8)		44 (25.1)	0.668	117 (21.7)
*Co-medication*								
PPI, n (%)	47 (56.6)	74 (54.8)	0.794		41 (41.8)	78 (38.6)	0.593	240 (44.5)
CCB, n (%)	23 (27.7)	28 (20.9)	0.250		6 (6.1)	0 (0.0)	0.000	57 (10.6)
Statin, n (%)	51 (61.4)	71 (53.0)	0.222		21 (21.4)	0 (0)	0.000	143 (26.5)
Morphine, n (%)	2 (2.4)	2 (1.5)	0.620		0 (0.0)	0 (0.0)	N/A	4 (0.7)
Platelet count (× 10^9^/L)	206.85 ± 77.01	202.28 ± 63.74	0.638		106.27 ± 109.76	177.87 ± 92.43	0.147	177.15 ± 94.81
300 mg of clopidogrel, n (%)	41 (45.6)	78 (52.3)	0.309		47 (48.8)	105 (52.0)	0.514	271 (50.3)
MPV (fL)	9.45 ± 3.79	9.29 ± 4.05	0.761		8.06 ± 4.73	9.20 ± 4.02	0.032	9.06 ± 4.15
PRI (%)	34.14 ± 11.42	67.41 ± 10.89	0.000		29.89 ± 14.33	73.09 ± 10.36	0.000	57.16 ± 21.90

PPI, proton pump inhibitor; CCB, calcium channel blocker; MPV, mean platelet volume

**Table 3:** Distribution genotypes and allele frequencies and the candidate SNPs between CR and non-CR patients

Gene/SNP	Genotype	Non-CR	CR	Co-dominat *P* value	Dominant *P* value	Recessive *P* value
CYP2C19*2	No. of patients with data	90	149	0.020	0.031	0.000
	*1/*1, n (%)	52 (57.8)	64 (43.0)			
	*1/*2, n (%)	37 (41.1)	74 (49.7)			
	*2/*2, n (%)	1 (1.1)	11 (7.4)			
CYP2C19*3	No. of patients with data	87	144	0.227	N/A	0.227
	*1/*1, n (%)	81 (93.1)	127 (88.2)			
	*1/*3, n (%)	6 (6.9)	17 (11.8)			
CYP2C19*2*3	No. of patients with data	87	144	0.072	N/A	0.072
	*1/*1, n (%)	46 (52.9)	53 (36.8)			
	*1/*2 + *1/*3, n (%)	7 (8.0)	2 (1.4)			
	*2/*2 + *1/*3, n (%)	0 (0.0)	0 (0.0)			
*P2RY12* rs2046934	No. of patients with data	87	141	0.945	N/A	0.945
	GG, n (%)	51 (58.6)	82 (58.2)			
	GA, n (%)	36 (41.4)	59 (41.8)			
*P2RY12* rs6809699	No. of patients with data	86	141	0.043	0.115	0.021
	CC, n (%)	76 (88.4)	107 (75.9)			
	CA, n (%)	10 (11.6)	30 (21.3)			
	AA, n (%)	0 (0)	4 (2.8)			
*PIK3CA* rs67562832	No. of patients with data	84	146	0.470	0.629	0.336
	AA, n (%)	64 (76.2)	119 (81.5)			
	AG, n (%)	19 (22.6)	24 (16.4)			
	GG, n (%)	1 (1.2)	3 (2.1)			
*PIK3CA* rs77576241	No. of patients with data	86	146	0.303	0.442	0.133
	CC, n (%)	84 (97.7)	136 (93.2)			
	CT, n (%)	2 (2.3)	9 (6.2)			
	TT, n (%)	0 (0)	1 (0.7)			
*APBB1IP* rs11015149	No. of patients with data	86	146	0.024	0.023	0.364
	CC, n (%)	73 (84.9)	117 (80.1)			
	CA, n (%)	10 (11.6)	29 (19.9)			
	AA, n (%)	3 (3.5)	0 (0)			
*TLN1* rs2295795	No. of patients with data	85	145	0.326	0.135	0.672
	GG, n (%)	47 (55.3)	76 (52.4)			
	GA, n (%)	36 (42.4)	59 (40.7)			
	AA, n (%)	2 (2.4)	10 (6.9)			
*TLN1* rs10814270	No. of patients with data	85	147	0.422	0.346	0.241
	CC, n (%)	21 (24.7)	47 (32.0)			
	CT, n (%)	44 (51.8)	73 (49.7)			
	TT, n (%)	20 (23.5)	27 (18.4)			
*ITGB3* rs3785873	No. of patients with data	85	145	0.236	0.634	0.090
	GG, n (%)	61 (71.8)	88 (60.7)			
	GA, n (%)	20 (23.5)	48 (33.1)			
	AA, n (%)	4 (4.7)	9 (6.2)			
*ITGB3* rs58847127	No. of patients with data	86	145	0.363	0.440	0.291
	GG, n (%)	71 (82.6)	127 (87.6)			
	GC, n (%)	15 (17.4)	17 (11.7)			
	CC, n (%)	0 (0)	1 (0.4)			
*ITGA2B* rs3760364	No. of patients with data	86	144	0.853	N/A	0.853
	TT (%)	80 (93)	133 (92.4)			
	TA (%)	6 (7)	11 (7.6)			
*RASGRP2* rs2230414	No. of patients with data	86	143	0.642	0.716	0.478
	CC, n (%)	39 (45.3)	58 (40.6)			
	CA, n (%)	36 (41.9)	69 (48.3)			
	AA, n (%)	11 (12.8)	16 (11.2)			

### Association of candidate SNPs with clopidogrel response

Genotype distribution of the 13 studied SNPs in the CR and non-CR groups were summarized in Table 3. Fitness to Hardy–Weinberg equilibrium was observed for each of the SNP (*P* > 0.05). The significant difference in genotype distribution for the CYP2C19*2 polymorphism (co-dominant *P* = 0.020, recessive *P* = 0.031, and dominant *P* = 0.000), the *P2Y12* rs6809699 polymorphism (co-dominant *P* = 0.043, recessive *P* = 0.115, and dominant *P* = 0.021) and the *APBB11P* rs11015149 polymorphism (co-dominant *P* = 0.024 and recessive *P* = 0.023) was observed between CR and non-CR patients (Table 3). However, the Benjamini–Hochberg adjusted *P* values is higher than the false discovery rate (0.05) except the dominant *P* Value of CYP2C19*2 (data not shown). Carriers of the CYP2C19*2 allele (57.0% vs 42.2%, CR vs non-CR, *P* = 0.026) and the *P2RY12* rs6809699 A allele (24.1% vs 14.0%, CR vs non-CR, *P* = 0.053) was obviously over-represented in the clopidogrel CR group. The frequency of carriers of the CYP2C19*3 allele trended to be increased in clopidogrel CR patients (11.8% vs 6.9%, CR vs non-CR, *P* = 0.227), though a significant difference was obtained. No difference in genotype distribution of other SNPs was observed between CR and non-CR groups (*P* > 0.05). And there was no association between the genetic polymorphisms and the occurrence of major adverse cardiovascular events (MACE) among the patients has been observed (Additional file 1: Table S1).

Unconditional logistic analysis was carried out for SNPs showed a significant difference in genotype distribution between clopidogrel CR and no-CR patients. After adjusted for dyslipidemia and concomitant use of statins and proton pump inhibitors, our results showed that patients with CYP2C19*2/*2 genotype showed significantly increased risk of CR (OR 7.406, 95% CI 0.894–61.361; *P* = 0.063) as compared with CYP2C19*1/*1homozygotes. When both *CYP2C19* LOF alleles (*2 and *3) were considered, CYP2C19 poor metabolizers (PMs, CYP2C19*2/*2 or CYP2C19*2/*3 or CYP2C19*3/*3) showed significantly increased risk of CR (OR 4.599, 95% CI 1.221–17.320, *P* = 0.024) as compared with wild-type homozygous for both SNPs. Patients carrying the *P2RY12* rs6809699 CA genotype or the rs6809699 A allele also showed significantly increased risk of CR (CA vs CC genotype: OR 2.270, 95% CI 1.019–5.059, *P* = 0.045; CA + AA vs CC genotype: OR 2.636, 95% CI 1.199–5.796; *P* = 0.016). The *APBB11P* rs11015149 polymorphism showed no association with clopidogrel response (*P* > 0.05). (Table 4).

**Table 4:** Logistic analysis of the association of gene polymorphisms and clopidogrel response

Gene/SNP	Genotype	Non-CR	CR	OR^a^ (95% CI)	*P*^a^ value
CYP2C19*2	*1/*1, n (%)	52 (57.8)	64 (43.0)	1.0 (ref)	N/A
	*1/*2, n (%)	37 (41.1)	74 (49.7)	1.625 (0.949–2.783)	0.076
	*2/*2, n (%)	1 (1.1)	11 (7.4)	8.938 (1.117–71.509)	0.015
	Carriers of *2	38 (42.2)	85 (57.0)	1.817 (1.070–3.086)	0.026
CYP2C19*3	*1/*1, n (%)	81 (93.1)	127 (88.2)	1.0 (ref)	N/A
	*1/*3, n (%)	6 (6.9)	17 (11.)	1.807 (0.684–4.774)	0.227
CYP2C19*2 and *3	*1/*1, n (%)	46 (52.9)	53 (36.8)	1.0 (ref)	N/A
	*1/*2 + *1/*3, n (%)	7 (8.0)	2 (1.4)	0.248 (0.049–1.253)	0.072
	*2/*2 + *1/*3, n (%)	0 (0.0)	0 (0.0)	4.509 (1.387–14.660)	0.012
	Carriers of *2 or *3, n (%)	7 (8.0)	2 (1.4)	0.248 (0.049–1.253)	0.072
*P2RY12* rs6809699	CC, n (%)	74 (86.0)	107 (75.9)	1.0 (ref)	N/A
	CA, n (%)	12 (14.0)	30 (21.3)	1.729 (0.831–3.595)	0.140
	AA, n (%)	0 (0)	4 (2.8)	N/A	0.099
	CA + AA, n (%)	12 (14.0)	35 (24.1)	2.017 (0.982–4.142)	0.053
*APBB1IP* rs11015149	CC, n (%)	73 (84.9)	117 (80.1)	1.0 (ref)	N/A
	CA, n (%)	10 (11.6)	29 (19.9)	1.809 (0.833–3.931)	0.130
	AA, n (%)	3 (3.5)	0 (0)	N/A	0.030
	CA + AA, n (%)	13 (15.1)	29 (19.9)	1.392 (0.680–2.850)	0.364

^a^Adjusted for use of statins and dyslipidemia

### Combined influence of CYP2C19 LOF and P2RY12 rs6809699 polymorphism on on-treatment PRI

**Fig. 1:**
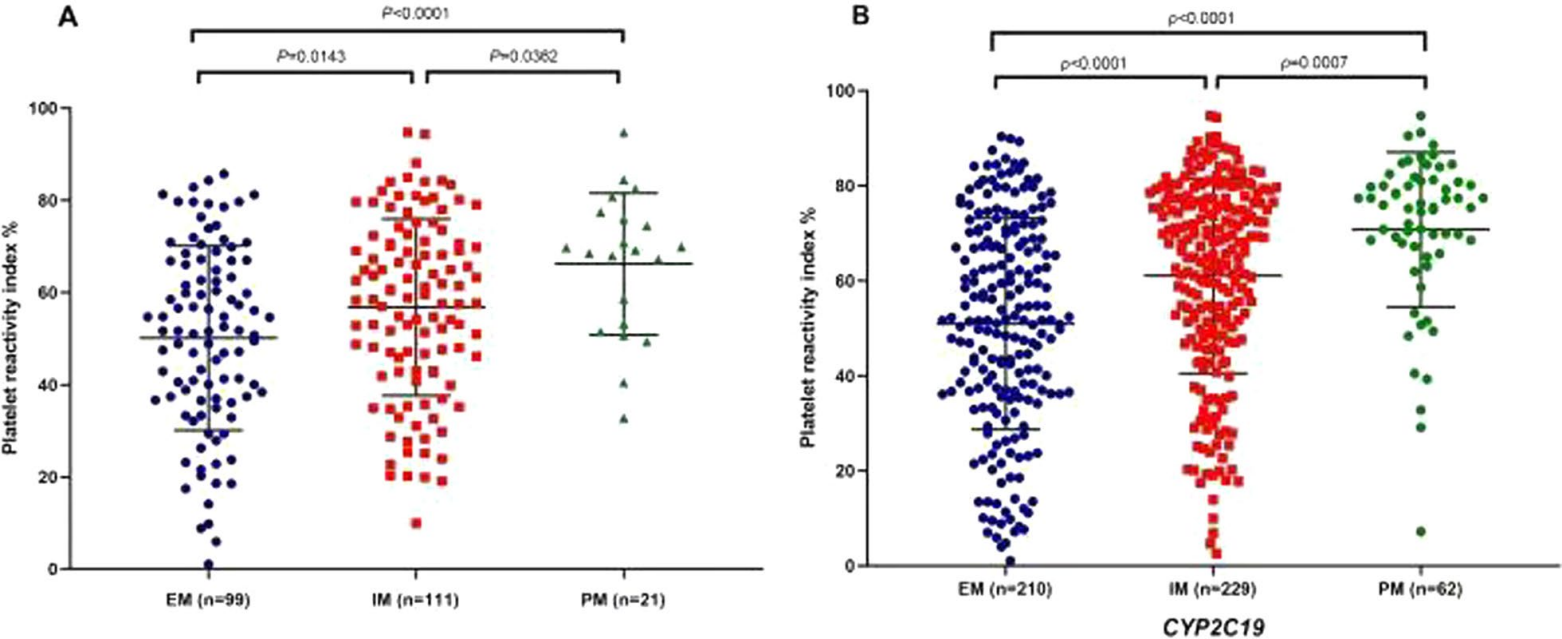
Comparison of platelet reactivity index (PRI) in CHD patients among CYP2C19*2 and CYP2C19*2 and *3 genotype groups in discovery samples (**A**) and all subjects (**B**)

Mean PRI among *CYP2C19* genotypes were shown in Fig. 1. Patients were grouped into EMs, IMs, and PMs according to carrying status of the CYP2C19*2 and CYP2C19*3 alleles. In the discovery samples and all samples, PM patients showed significantly higher PRI than IM and EM patients, respectively. The influence of CYP2C19*2 polymorphism on PRI was observed, which was not found in CYP2C19*3 polymorphism (Table 5).

**Table 5:** Comparison of platelet reactivity index (PRI) in CHD patients among CYP2C19*2 and *3 genotype groups

SNP	Genotype	PRI (Discovery samples)	*P* value	PRI (All samples)	*P* value
CYP2C19*2	GG	51.27 ± 19.69	0.0015	51.94 ± 22.10	< 0.0001
	AG	56.99 ± 19.02		61.74 ± 20.08	
	AA	70.33 ± 13.47		72.18 ± 15.71	
CYP2C19*3	GG	54.21 ± 20.01	0.1719	57.64 ± 21.98	0.1742
	AG	60.15 ± 16.50		61.05 ± 20.17	
	AA	–		82.71 ± 11.00	

**Fig. 2:**
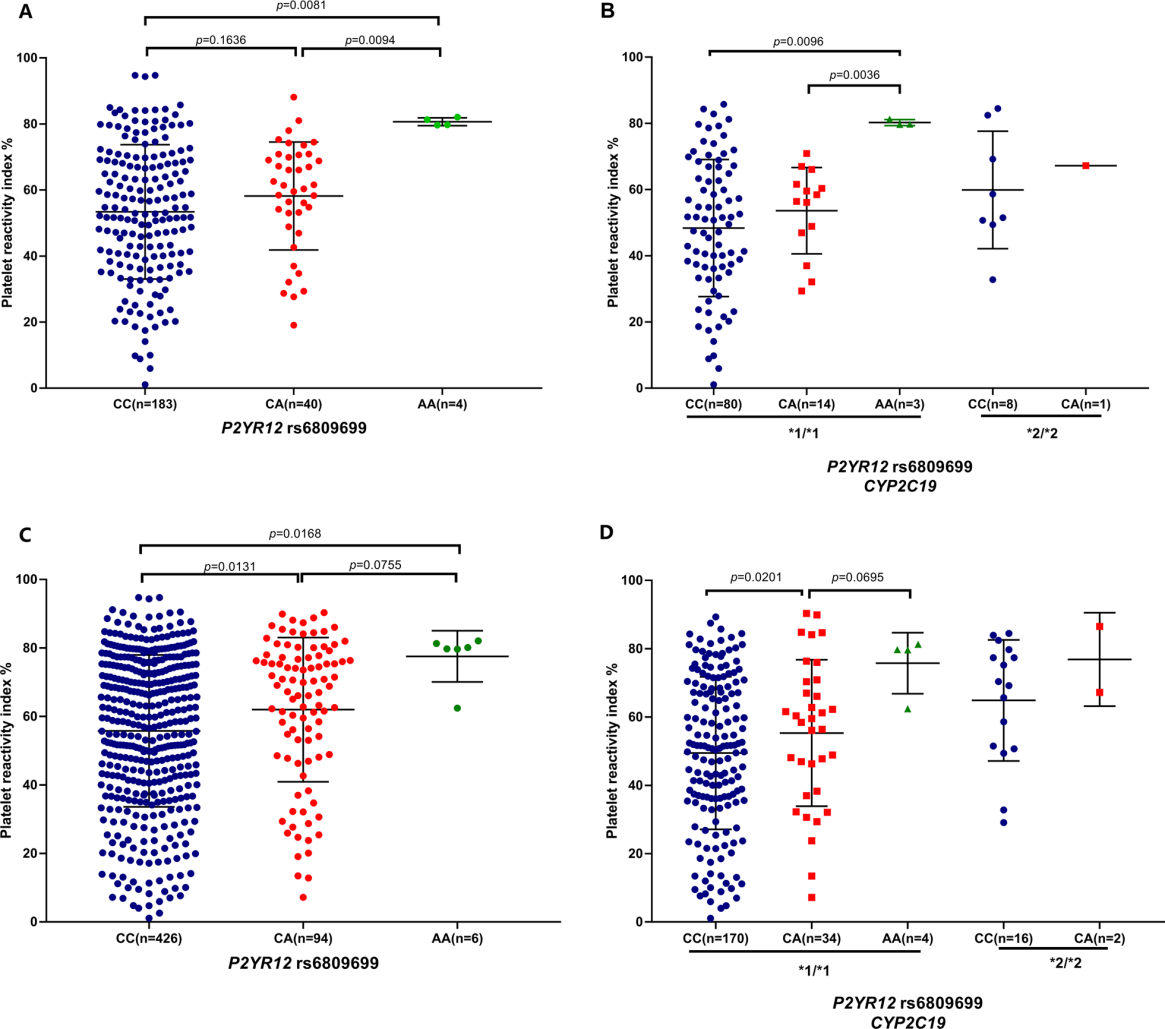
Comparison of PRI in CHD patients among *P2RY12* rs6809699 genotypes stratified by *CYP2C19* genotypes in discovery samples (**A**, **B**) and all subjects (**C**, **D**)

For the *P2RY12* rs6809699 genotypes, these patients with mutant homozygous AA (n = 4) were showed significantly higher PRI than the wild-type CC (n = 183) and heterozygous CA (n = 40) genotype groups (*P* = 0.0081 and 0.0094, respectively, Fig. 2A). In consideration that the influence of *P2RY12* rs6809699 on clopidogrel response might be affected by *CYP2C19* LOF, stratification analysis according to *CYP2C19* genotypes was further performed. As shown in Fig. 2A, rs6809699 AA homozygotes showed significantly higher PRI than patients carrying both the rs6809699 CC and the rs6809699 CA genotypes (*P* = 0.0096 and 0.0036, respectively). Only one patient with the AA genotype in carriers of the *CYP2C19* LOF limited statistical analysis in these patients, but the tendency remained. Then the rs6809699 was validated in all subjects (discovery and validation samples) (Fig. 2).

## Discussion

In this study, we evaluated the effects of genetic polymorphisms in the P2Y12 receptor-mediated signaling pathway and *CYP2C19* on clopidogrel antiplatelet response in Chinese CHD patients. We observed that CYP2C19*2 and *3 and *P2Y12* rs6809699 polymorphisms were associated with an increased risk of clopidogrel resistance indicated by platelet VASP-P level.

Clopidogrel is a prodrug that needs to be bioactivated in two sequential cytochrome P450-dependent steps before it exerts an inhibitory effect on ADP-induced platelet aggregation. According to the literature, the prevalence of clopidogrel resistance among the Asian population was estimated at 17.2–81.6% [26]. In this study, a total of 539 consecutive Chinese patients with coronary heart disease were recruited and found that 65.1% patients had clopidogrel resistance. CYP2C19 activity is reported to be crucial in the metabolism and efficacy of clopidogrel. *CYP2C19* LOF alleles, including *2 and *3 can decrease the plasma concentration and AUC_0–24 h_ of the active metabolite of clopidogrel, which results in impaired antiplatelet effect of clopidogrel [6, 24]. A recent meta-analysis has concluded that *CYP2C19* LOF is associated with increased risk of adverse clinical events in patients who underwent clopidogrel therapy despite differences in clinical significance according to ethnicity [7]. In support of these previous reports, we observed that patients with the CYP2C19*1/*1 genotype showed significantly lower PRI than the CYP2C19*2 heterozygous and homozygous genotypes. Besides, we found that carriers of any of the *2 and *3 alleles showed increased clopidogrel resistance. Our findings further confirmed the pivotal role of CYP2C19*2 and *3 as pharmacogenomics markers for clopidogrel response. In the 2013 updated Clinical Pharmacogenetics Implementation Consortium Guidelines for *CYP2C19* Genotype and Clopidogrel Therapy, *CYP2C19* genotype-guided clopidogrel therapy was recommended to ACS patients underwent PCI [27]. Standard dosing of clopidogrel is warranted among ACS/PCI patients with a predicted CYP2C19 extensive metabolizer phenotype (*1/*1). If genotyping identifies a patient as a CYP2C19 weak metabolizer phenotype (*2/*2, *2/*3 and *3/*3), the use of an alternative antiplatelet agent (e.g., prasugrel or ticagrelor) is recommended if not clinically contraindicated.

ADP is an essential activator of platelet and acts via P2Y1 (Gq-coupled) and P2Y12 (Gi-coupled) receptors. The Gq-coupled P2Y1 receptor is vital in Ca^2+^ mediated platelet shape change, while the Gi-coupled P2Y12 receptor is required for ADP-induced platelet activation [28]. The active metabolite of clopidogrel binds to the P2Y12 receptor irreversibly and inhibits ADP-mediated platelet activation and aggregation. The role of the *P2RY12* genetic polymorphisms in clopidogrel response has been assessed previously [19, 20, 29–32]. Evidence shows that the *P2RY12* T744C (rs2046934) polymorphism is associated with enhanced platelet aggregation and increased risk of atherothrombosis [19, 30]. However, Thomas et al. failed to replicate this observation with platelet activity assessed by either ADP-Ag (*P* = 0.39), or PRI VASP-P (*P* = 0.97), or P-selectin expression (*P* = 0.62) in 597 NSTE ACS patients [27]. Other studies also come to negative findings [31, 32]. In agreement with the latter investigators, we did not find any association between the *P2RY12* T744C and clopidogrel resistance either*.*

The *P2RY12* G52T (rs6809699) was also shown to be associated with increased risk of clopidogrel resistance and cardiovascular events in Chinese ACS patients after PCI [20]. In support of this report, we observed that CHD patients with the *P2RY12* rs6809699 CA genotype or carriers of the rs6809699 A allele showed an increased risk for clopidogrel resistance with an OR of 1.729 and 2.017, respectively. After stratification by CYP2C19*2 and *3 carrying status, the *P2RY12* rs6809699 polymorphism remained to be associated with increased platelet activity. As the rs6809699 polymorphism is a synonymous SNP (Gly12Gly) does not result in amino acid change, the exact function of this SNP deserved further investigation.

Abnormality in GPIIb/IIIa complex is reported in Glanzmann’s thrombasthenia patients with impaired platelet aggregation and increased bleeding [33]. The *ITGB3* PlA1/A2 polymorphism (rs5918) results in a leucine (PlA1) to proline (PlA2) substitution in exon2 was observed [34]. This SNP has been extensively studied and is shown to be associated with both antiplatelet drug resistance and increased cardiovascular events [35, 36]. Because the prevalence of the PlA2 allele is low in the Chinese population, the SNP was not included in our study. Two other SNPs, including rs3785873 and rs58847127 at the *ITGB3* locus were investigated in our study. However, no significant findings were obtained for these two SNPs. A healthy subjects study showed that *ITGA2B* rs3760364 were related to bleeding time [17], but we failed to find the association between *ITGA2B* rs3760364 and platelet activity.

In our study, we also observed that the *APBB11P* rs11015149 A allele was significantly over-represented in CR than non-CR patients, but this difference was disappeared after adjusted for statins use and dyslipidemia. The other 6 selected SNPs in genes in the P2Y12-mediated signaling pathway (*PIK3CA* rs67562832 and rs67562832*, RASGRP2* rs2230414*, APBB1IP* rs11015149*, TLN1* rs2295795, and rs10814270) also showed no association with clopidogrel resistance. It remains unknown whether genetic factors in other alternative pathways playing compensatory roles in GPIIb/IIIa inside-out signaling could affect clopidogrel response.

Although the *CYP2C19* genotyping had been widely recommended when considering clopidogrel for cardiovascular indications, it remains undetermined that *P2RY12* polymorphisms associated with clopidogrel resistance. In our study, we reconfirmed the impact of CYP2C19*2, *3 and *P2RY12* rs6809699 polymorphisms on impaired antiplatelet effects of clopidogrel in Chinese CHD patients. It suggested that *P2RY12* genetic polymorphisms may serve as biomarkers for clopidogrel response. Meanwhile, we found the increased risk of clopidogrel resistance in CYP2C19*1/*1 homozygous who carrying the *P2RY12* rs6809699 A allele. This may, at least partially, explain that some CYP2C19 CYP2C19*1/*1 homozygous were still resistant to clopidogrel. Therefore, construction of a comprehensive prediction model of clopidogrel responsiveness based on clinical factors and multiple gene polymorphisms, including CYP2C19 and *P2RY12* polymorphisms, has more clinical significance for guiding the precise medication of clopidogrel.

Limitations of the study include a relatively small sample size. As exemplified by only 4 patients with *P2RY12* rs6809699 mutant AA genotype in our study, further studies are warranted to verify the impact of *P2RY12* rs6809699 polymorphisms on antiplatelet effects of clopidogrel. Secondly, platelet function testing was done with only a single assessment of platelet function, VASP-P assay, which may not be sufficient to fully reflect the response to antiplatelet therapy. Finally, follow-up data is warranted to understand the influence of the positively associated SNPs on the endpoint events and outcome of CHD patients with long-time clopidogrel therapy.

## Conclusions

This study confirms the impact of CYP2C19*2, *3 and *P2RY12* rs6809699 polymorphisms on impaired antiplatelet effects of clopidogrel in Chinese CHD patients. Moreover, the influence of *P2RY12* rs6809699 on clopidogrel response is independent of *CYP2C19* LOF alleles. But SNPs in other genes in the P2Y12 receptor pathway were not associated with antiplatelet effects of clopidogrel. A study with a larger sample size is required to confirm the association of the *P2RY12* rs6809699 with adverse ischemic events in patients receiving clopidogrel therapy. And also, the exact function of *P2RY12* rs6809699 on P2Y12 expression or function is needed.

## Supplementary Information

**Additional file 1: Table S1**. Distribution genotypes and allele frequencies and the candidate SNPs between patients with and without MACE.

## Data Availability

The first author can be contacted if the raw data are needed. The email address of the first author is dongjieli@csu.edu.cn.

## References

[1] Benjamin EJ, Blaha MJ, Chiuve SE, Cushman M, Das SR, Deo R (2017). Heart disease and stroke statistics-2017 update: a report from the American Heart Association. Circulation.

[2] Jaremo P, Lindahl TL, Fransson SG, Richter A (2002). Individual variations of platelet inhibition after loading doses of clopidogrel. J Intern Med.

[3] Matetzky S, Shenkman B, Guetta V, Shechter M, Beinart R, Goldenberg I (2004). Clopidogrel resistance is associated with increased risk of recurrent atherothrombotic events in patients with acute myocardial infarction. Circulation.

[4] Zhang YJ, Li MP, Tang J, Chen XP (2017). Pharmacokinetic and pharmacodynamic responses to clopidogrel: evidences and perspectives. Int J Environ Res Public Health.

[5] Mega JL, Close SL, Wiviott SD, Shen L, Hockett RD, Brandt JT (2009). Cytochrome p-450 polymorphisms and response to clopidogrel. N Engl J Med.

[6] Djebli N, Fabre D, Boulenc X, Fabre G, Sultan E, Hurbin F (2015). Physiologically based pharmacokinetic modeling for sequential metabolism: effect of CYP2C19 genetic polymorphism on clopidogrel and clopidogrel active metabolite pharmacokinetics. Drug Metab Dispos.

[7] Jang JS, Cho KI, Jin HY, Seo JS, Yang TH, Kim DK (2012). Meta-analysis of cytochrome P450 2C19 polymorphism and risk of adverse clinical outcomes among coronary artery disease patients of different ethnic groups treated with clopidogrel. AM J Cardiol.

[8] Li Z, Delaney MK, O'Brien KA, Du X (2010). Signaling during platelet adhesion and activation. Arterioscler Thromb Vasc Biol.

[9] Springer TA, Dustin ML (2012). Integrin inside-out signaling and the immunological synapse. Curr Opin Cell Biol.

[10] Li Z, Zhang G, Le Breton GC, Gao X, Malik AB, Du X (2003). Two waves of platelet secretion induced by thromboxane A2 receptor and a critical role for phosphoinositide 3-kinases. J Biol Chem.

[11] Lova P, Paganini S, Hirsch E, Barberis L, Wymann M, Sinigaglia F (2003). A selective role for phosphatidylinositol 3,4,5-trisphosphate in the Gi-dependent activation of platelet Rap1B. J Biol Chem.

[12] Consonni A, Cipolla L, Guidetti G, Canobbio I, Ciraolo E, Hirsch E (2012). Role and regulation of phosphatidylinositol 3-kinase beta in platelet integrin alpha2beta1 signaling. Blood.

[13] Crittenden JR, Bergmeier W, Zhang Y, Piffath CL, Liang Y, Wagner DD (2004). CalDAG-GEFI integrates signaling for platelet aggregation and thrombus formation. Nat Med.

[14] Lagarrigue F, Kim C, Ginsberg MH (2016). The Rap1-RIAM-talin axis of integrin activation and blood cell function. Blood.

[15] Lee HS, Lim CJ, Puzon-McLaughlin W, Shattil SJ, Ginsberg MH (2009). RIAM activates integrins by linking talin to ras GTPase membrane-targeting sequences. J Biol Chem.

[16] Canault M, Ghalloussi D, Grosdidier C, Guinier M, Perret C, Chelghoum N (2014). Human CalDAG-GEFI gene (RASGRP2) mutation affects platelet function and causes severe bleeding. J Exp Med.

[17] Xiang Q, Ji SD, Zhang Z, Zhao X, Cui YM (2016). Identification of ITGA2B and ITGB3 single-nucleotide polymorphisms and their influences on the platelet function. Biomed Res Int.

[18] Li MP, Xiong Y, Xu A, Zhou JP, Tang J, Zhang ZL (2014). Association of platelet ITGA2B and ITGB3 polymorphisms with ex vivo antiplatelet effect of ticagrelor in healthy Chinese male subjects. Int J Hematol.

[19] Fontana P, Dupont A, Gandrille S, Bachelot-Loza C, Reny JL, Aiach M (2003). Adenosine diphosphate-induced platelet aggregation is associated with P2Y12 gene sequence variations in healthy subjects. Circulation.

[20] Li M, Wang H, Xuan L, Shi X, Zhou T, Zhang N (2017). Associations between P2RY12 gene polymorphisms and risks of clopidogrel resistance and adverse cardiovascular events after PCI in patients with acute coronary syndrome. Medicine (Baltimore).

[21] Valgimigli M (2021). P2Y12 inhibitor monotherapy or dual antiplatelet therapy after coronary revascularisation: individual patient level meta-analysis of randomised controlled trials. BMJ (Clin Res ed.).

[22] Bonello L, Paganelli F, Arpin-Bornet M, Auquier P, Sampol J, Dignat-George F (2007). Vasodilator-stimulated phosphoprotein phosphorylation analysis prior to percutaneous coronary intervention for exclusion of postprocedural major adverse cardiovascular events. J Thromb Haemost.

[23] Wang G, Lei HP, Li Z, Tan ZR, Guo D, Fan L (2009). The CYP2C19 ultra-rapid metabolizer genotype influences the pharmacokinetics of voriconazole in healthy male volunteers. Eur J Clin Pharmacol.

[24] Lin R, Zhang L, Zhang P, Zhou L, Liu T, Li Y (2015). Influence of CYP2C19 loss-of-function variants on the metabolism of clopidogrel in patients from north-western China. J Clin Pharm Ther.

[25] Hoshino K, Horiuchi H, Tada T, Tazaki J, Nishi E, Kawato M (2009). Clopidogrel resistance in Japanese patients scheduled for percutaneous coronary intervention. Circ J.

[26] Akkaif M, Daud N, Sha’aban A (2021). The role of genetic polymorphism and other factors on clopidogrel resistance (CR) in an Asian Population with Coronary Heart Disease (CHD). Molecules (Basel, Switzerland).

[27] Scott SA, Sangkuhl K, Stein CM, Hulot JS, Mega JL, Roden DM (2013). Clinical pharmacogenetics implementation consortium guidelines for CYP2C19 genotype and clopidogrel therapy: 2013 update. Clin Pharmacol Ther.

[28] Jin J, Kunapuli SP (1998). Coactivation of two different G protein-coupled receptors is essential for ADP-induced platelet aggregation. Proc Natl Acad Sci U S A.

[29] Cuisset T, Frere C, Quilici J, Morange PE, Saut N, Lambert M (2007). Role of the T744C polymorphism of the P2Y12 gene on platelet response to a 600-mg loading dose of clopidogrel in 597 patients with non-ST-segment elevation acute coronary syndrome. Thromb Res.

[30] Staritz P, Kurz K, Stoll M, Giannitsis E, Katus HA (2009). Platelet reactivity and clopidogrel resistance are associated with the H2 haplotype of the P2Y12-ADP receptor gene. Int J Cardiol.

[31] Kar R, Meena A, Yadav BK, Yadav R, Kar SS, Saxena R (2013). Clopidogrel resistance in North Indian patients of coronary artery disease and lack of its association with platelet ADP receptors P2Y1 and P2Y12 gene polymorphisms. Platelets.

[32] Sun B, Li J, Dong M, Yang L, Wu C, Zhu L (2015). Diversity of platelet function and genetic polymorphism in clopidogrel-treated Chinese patients. Genet Mol Res.

[33] Bellucci S, Caen J (2002). Molecular basis of Glanzmann's thrombasthenia and current strategies in treatment. Blood Rev.

[34] Maguire J, Thakkinstian A, Levi C, Lincz L, Bisset L, Sturm J (2011). Impact of COX-2 rs5275 and rs20417 and GPIIIa rs5918 polymorphisms on 90-day ischemic stroke functional outcome: a novel finding. J Stroke Cerebrovasc Dis.

[35] Angiolillo DJ, Fernandez-Ortiz A, Bernardo E, Alfonso F, Sabate M, Fernandez C (2004). PlA polymorphism and platelet reactivity following clopidogrel loading dose in patients undergoing coronary stent implantation. Blood Coagul Fibrinolysis.

[36] Floyd CN, Ellis BH, Ferro A (2014). The PlA1/A2 polymorphism of glycoprotein IIIa as a risk factor for stroke: a systematic review and meta-analysis. PLoS ONE.

