# Supplementary material for: Influence of genetic polymorphisms in P2Y12 receptor signaling pathway on antiplatelet response to clopidogrel in coronary heart disease
Source: BMC Cardiovasc Disord. 2022 Dec 30;22:575. doi: 10.1186/s12872-022-02988-w (PMC9801627; doi:10.1186/s12872-022-02988-w)
Supplement: Supplementary file 1 — Additional file 1: Table S1. Distribution genotypes and allele frequencies and the candidate SNPs between patients with and without MACE. [file 12872_2022_2988_MOESM1_ESM.docx]

| **Gene/SNP** | **Genotype** | **Patients without MACE** | **Patients**  **with MACE** | **Co-dominat**  **P Value** | **Recessive**  **P Value** | **Dominant**  **P Value** |
| --- | --- | --- | --- | --- | --- | --- |
| *CYP2C19*2* | No. of patients with data | 45 | 12 | 0.125 | 0.211 | 0.731 |
|  | *1/*1, n (%) | 20 (44.4) | 6 (50.0) |  |  |  |
|  | *1/*2, n (%) | 25 (55.6) | 5 (41.7) |  |  |  |
|  | *2/*2, n (%) | 0 (0) | 1 (8.3) |  |  |  |
| *CYP2C19*3* | No. of patients with data | 43 | 28 | 0.746 | N/A |  |
|  | *1/*1, n (%) | 38 (88.4) | 11 (39.2) |  |  | 0.746 |
|  | *1/*3, n (%) | 5 (11.6) | 17 (60.7) |  |  |  |
| *P2RY12* rs6809699 | No. of patients with data | 41 | 12 | 0.168 | 0.226 | 0.400 |
|  | CC, n (%) | 35 (85.4) | 9 (75.0) |  |  |  |
|  | CA, n (%) | 6 (14.6) | 2 (16.7) |  |  |  |
|  | AA, n (%) | 0 (3.4) | 1 (12.5) |  |  |  |
| *APBB1IP* rs11015149 | No. of patients with data | 45 | 11 |  |  |  |
|  | CC, n (%) | 39 (86.7) | 10 (90.9) | 0.862 | 0.618 | 0.703 |
|  | CA, n (%) | 5 (11.1) | 1 (9.1) |  |  |  |
|  | AA, n (%) | 1 (2.2) | 0 (0) |  |  |  |

**Supplemental Table 1.** Distribution genotypes and allele frequencies and the candidate SNPs between patients with and without MACE.
